# Safety and effectiveness of stem cell therapies in early-phase clinical trials in stroke: a systematic review and meta-analysis

**DOI:** 10.1186/s13287-017-0643-x

**Published:** 2017-08-30

**Authors:** Anjali Nagpal, Fong Chan Choy, Stuart Howell, Susan Hillier, Fiona Chan, Monica A. Hamilton-Bruce, Simon A. Koblar

**Affiliations:** 1 Stroke Research Programme, The University of Adelaide School of Medicine, Level 6 South, SAHMRI, North Terrace, Adelaide, South Australia Australia; 2 Data, Design and Statistics Service, Adelaide Health Technology Assessment (AHTA), School of Public Health, The University of Adelaide, Adelaide, South Australia Australia; 3 Research, Sansom Institute for Health Research, University of South Australia, Adelaide, South Australia Australia; 4 Neurology Department, The Queen Elizabeth Hospital, Central Adelaide Local Health Network (CALHN), Adelaide, South Australia Australia; 5 Department of Neurology, Royal Adelaide Hospital, CALHN, Adelaide, South Australia Australia

**Keywords:** Stem cells, Stroke, Clinical design, Outcomes, Regenerative medicine

## Abstract

Stem cells have demonstrated encouraging potential as reparative therapy for patients suffering from post-stroke disability. Reperfusion interventions in the acute phase of stroke have shown significant benefit but are limited by a narrow window of opportunity in which they are beneficial. Thereafter, rehabilitation is the only intervention available. The current review summarises the current evidence for use of stem cell therapies in stroke from early-phase clinical trials. The safety and feasibility of administering different types of stem cell therapies in stroke seem to be reasonably proven. However, the effectiveness needs still to be established through bigger clinical trials with more pragmatic clinical trial designs that address the challenges raised by the heterogeneous nature of stroke per se, as well those due to unique characteristics of stem cells as therapeutic agents.

**Electronic supplementary material:**

The online version of this article (doi:10.1186/s13287-017-0643-x) contains supplementary material, which is available to authorized users.

## Background

Stroke, classically characterised as a neurological deficit attributed to an acute focal injury of the central nervous system (CNS) by a vascular cause (infarction or haemorrhage), is a major cause of disability and death worldwide [1]. While stroke represents a single event of cell/tissue injury, it sets in motion a complex interplay of inflammation and repair involving neural, vascular and connective tissues, in and around the affected areas of the brain [2, 3]. Molecular and imaging research is generating new insights into mechanistic interactions at the cellular level [3]. The American Heart Association/American Stroke Association (AHA/ASA) proposed an updated definition for stroke in 2013 that incorporates clinical and tissue criteria [4]. These criteria reflect the advances in imaging techniques and consequent understanding of disease pathophysiology in the past few decades. However, the translation of these advances into meaningful therapeutic options has until recently been met with limited success. While interventions for early reperfusion such as thrombolysis and endovascular revascularisation have shown significant benefit, they are still subject to a limited window of opportunity [5, 6].

There is now a significant body of evidence from pre-clinical research which postulates that stem cells potentially modulate multiple pathways involved in endogenous neurogenesis, angiogenesis, immune modulation and neural plasticity, in addition to or instead of cell replacement [7–9]. These effects may potentially be harnessed for effecting structural and functional regeneration after stroke with a prolonged window of opportunity [10, 11]. An encouraging number of pilot and definitive early-phase clinical studies have been published in the last decade, signalling a critical milestone in clinical translation of stem cell therapies in stroke [12–39]. However, the interpretation of current knowledge in stem cell research seems challenging due to heterogeneity in the study design, publication bias and the possible confounding effects of concomitant interventions such as immunosuppressant use and rehabilitation. Early meta-analyses have attempted to investigate efficacy and safety data for particular cell types (e.g. mesenchymal cells) [40], stroke type (ischaemic) [41] or study design (single-arm studies) [42]. However, these analyses have been limited by the small number and size of studies considered. Other reviewers have taken more of an ‘all-comers’ approach to inclusion of all potential regenerative interventions including combinations of cell-based and biological therapies [43]. This approach, while attractive on a broader pathophysiological level, may present over-simplified assessment of the complexities involved in the use and investigation of living cells as therapeutic products.

### Objectives

The present review and meta-analysis aims to assess the effectiveness and safety of cell therapies, studied as a monotherapy (inclusive of any type/source/route of administration) in adult patients with stroke (inclusive of all types and phases of stroke) and published in English.

## Methods

### Protocol and registration

The protocol for the review was prepared and registered on PROSPERO (international prospective register of systematic reviews) [Ref-2016:CRD42016039524], and is available online (http://www.crd.york.ac.uk/PROSPERO/display_record.asp?ID=CRD42016039524).

### Eligibility criteria

The review evaluated all studies investigating the use of stem cells in stroke, other than case reports, reported in the English language during the period 2005–2016. The included studies were segregated into two subgroups for further analysis: controlled studies with a comparator arm and studies without comparator arms.

#### Inclusion criteria

Trials investigating the use of stem cell therapy in adult patients who had experienced a stroke, inclusive of all types of stroke and in any phase from the acute to chronic phase, were included.

#### Exclusion criteria

Trials investigating combination therapies including stem cells with other therapies.

### Intervention(s) of interest

Stem cell-based interventions with any type (autograft, allograft or xenograft; embryonic, fetal or adult) of cell source, route of administration (intracerebral/intravenous/intra-arterial/intrathecal) and dosage.

### Search strategy

Databases including PubMed, EMBASE, SCOPUS, Web of Science and the Cochrane Central Register of Controlled Trials (CENTRAL) registry of the Cochrane Collaboration were searched until November 2016. The specific search strategies were created in consultation with a health sciences librarian with expertise in systematic review searches. After the PubMed strategy was finalised, it was adapted to the syntax and subject headings of the other databases. The International Clinical Trials Registry Platform Search Portal and ClinicalTrials.gov were also searched for trials completed recently. AN, FC and FCC checked for additional relevant articles. The authors of articles were contacted via email when pertinent information was missing in the published manuscripts and additional data thus obtained were included in the final analysis (Additional file 1).

### Study selection

All studies identified using the search strategy described were screened independently by two review authors (AN and FCC). AN and FCC independently assessed full texts of all eligible studies. Any disagreement regarding the eligibility of a particular study was resolved through discussion with senior reviewers (SAK and SHi).

### Data collection process and data items

A standardised data extraction form was used to extract data from the included studies for assessment of study quality and evidence synthesis. The data extraction form was designed in consultation with the methodologist on the team (SHo). Extracted information included: study setting (year of publication and country); study population demographics and baseline characteristics; details of the intervention and control conditions, if applicable; recruitment and study completion rates; information for assessment of the risk of bias; and study design elements such as randomisation, blinding, treatment allocation and interventions in the control group, outcomes and times of measurement.

Two review authors (AN, FCC) extracted data independently and differences identified were resolved through discussion with senior reviewers (SAK, SHi, AHB). Study authors were contacted via email for missing data.

Characteristics of study participants such as demographic characteristics, the phase and type of stroke, time between stroke onset and enrolment, time between stroke onset and administration of stem cell therapy and delivery of rehabilitation were recorded. The outcomes assessed in different trials for determining safety, efficacy and feasibility were recorded along with the period of follow-up used in different studies.

### Risk of bias (quality) assessment

AN, FCC and SHi assessed the risk of bias in individual studies for the two subgroups included in the review, considering the characteristics recommended by the International Cochrane Collaboration [44].

### Summary measures

#### Primary outcomes

Primary outcomes of interest were based on the WHO ICF framework [45] and included effectiveness measures assessed at the 6-month time point using validated scales for body structure/impairment measures (e.g. National Institutes of Health Stroke Scale (NIHSS)/Fugyl-Meyer assessment/Modified Ashworth Scale/European Stroke Scale), activity measures (e.g. Barthel index (BI)) and participation measures (e.g. Stroke Impact Scale/Modified Rankin scale (mRS)).

#### Secondary outcomes

Post-procedure safety outcomes such as death, infections, stroke recurrence and neoplasms were considered. The minimum period of follow-up was established as 6 months.

### Strategy for data synthesis

A narrative synthesis of the findings from the included studies was carried out structured around the type of intervention, the study design, the target population characteristics and the effectiveness and safety outcomes measured. The included studies were segregated depending on study design into two subgroups for further analysis: controlled studies with a comparator arm and studies without comparator arms.

### Method for meta-analysis

The data were analysed by SHo using STATA/SE v14.1 (StataCorp LP, College Station, TX, USA).

For the single-arm studies, patient data were used to calculate a difference score, which represents the change from baseline to day 180. Meta-analyses were performed using only the mean and 95% confidence limits of the difference scores. Data from the controlled studies were explored using treatment effects (treatment vs control group) at 6 months. Baseline data for each study were inspected to ensure that the randomisation produced groups which did not differ in terms of mean scores for the three scales under investigation (NIHSS, BI and mRS), for which there were adequate data available. For both subgroups, a separate meta-analysis was performed for each instrument. The meta-analyses were performed using a DerSimonian–Laird random effects model to account for potential heterogeneity across studies [46]. Pooled estimates were presented as the standardised mean difference (SMD) with 95% confidence intervals. Heterogeneity was summarised using the *I*-squared statistic.

A formal evaluation of heterogeneity and publication bias was planned in the event of data being available from an adequate number of studies.

## Results

### Study selection

The review followed the Preferred Reporting Items for Systematic Reviews and Meta-Analyses (PRISMA) guidelines [47].

**Fig. 1:**
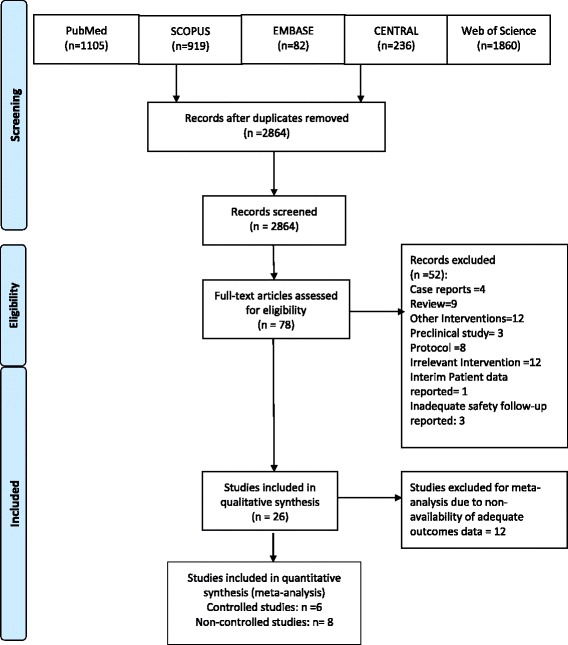
Study Selection for Review & Meta-analysis

Twenty-six studies, which fulfilled the defined inclusion and exclusion criteria, were selected for further data synthesis (Fig. 1).

### Study characteristics

The type of stem cell intervention (cell type; source; route of administration; time between stroke onset and administration of stem cell therapy; delivery of rehabilitation), study design and target population characteristics (phase and type of stroke; time between stroke onset and enrolment) are presented in Table 1.

**Table 1:** Disposition of study design & intervention characteristics

Study variable	Number of studies	Number of subjects
Studies included	26	844
Country		
United States	5	45
United Kingdom	2	16
Brazil	3	43
China	4	366
India	4	195
South Korea	2	82
Japan	2	24
Spain	1	10
Taiwan	1	30
Cuba	1	5
Russia	1	10
Stroke phase		
Hyper-acute/acute/sub-acute	15	597
Chronic	12	247
Stroke type		
Ischaemic	20	421
Ischaemic + haemorrhagic	5	77
Haemorrhagic	2	346
Stem cell characteristics		
Cell type		
Human bone marrow-derived MSC/MNC	18	698
Human fetal neural stem/progenitor cells	3^a^	67
Umbilical mesenchymal stem cells	1	14
Porcine fetal cells	1	5
Human embryonic neuronal cells	2	19
Peripheral blood haematopoietic stem cells	1	30
Cell source		
Allogeneic	8	134
Autologous	18	710
Route of administration		
Intra-arterial	6^b^	57
Intracerebral	9^c^	405
Intravenous	11^b^	332
Intrathecal	2^c^	256
Time between stroke onset and stem cell transplantation		
< 3 months	13^d^	567
> 3 months	14^d^	283
Study design		
RCT	6	390
Non-RCT (case–control design)	4	280
Non-RCT (historic control)	1	10
Single-arm open-label design	15	97
Provision of rehabilitation		
RCT		
Yes	4	
No	1	
Not reported	1	
Non-RCT		
Yes	1	
No	4	
Not reported	14	

*MSC* mesenchymal stem cells, *MNC* mononuclear cells, *RCT* randomised controlled trial

^a^Co-transplantation of neural stem cells and umbilical cord MSC

^b^One study had two unmatched sequential cohorts investigated under different routes of administration

^b^A treatment cycle in one study used transplants via intracerebral route followed by 4 weeks of intravenous infusion

^d^One study reported administration of stem cell therapy in two settings (1st setting before and 2nd after 3 months of stroke)

The majority of studies (n=18) utilised autologous adult human bone marrow-derived mesenchymal/mononuclear cells [13, 16–22, 24-26, 30–34, 36, 37, 39]. The remaining studies utilised varied allogeneic cell sources such as human neural stem cells derived from fetal tissue (n=3) [15, 29, 38], mesenchymal cells from umbilical cord blood (n=1) [23], neuronal cells derived from embryonic tissue (*n* = 2) [12, 14] and autologous peripheral blood haematopoietic stem cells (*n* = 1). One study investigated the use of a xenograft (porcine fetal cells; *n* = 1) [16].

The most common route of delivery of stem cells was intravenous (*n* = 11) followed by intracerebral (*n* = 9), intra-arterial (*n* = 6) and intrathecal (*n* = 2).

#### Studies without a comparator arm

Fifteen studies were evaluated in the single-arm study subgroup (*N*_experimental_ = 131). Ninety-six participants received stem cell transplantation within 3 months of the incident stroke, of which only one patient had a haemorrhagic stroke. Seventy-nine patients received stem cell transplantation more than 3 months post stroke, of which 65 participants had ischaemic stroke and two patients had haemorrhagic stroke.

#### Studies with a comparator arm

Eleven studies were evaluated in the controlled study subgroup (*N*_*experimental*_ = 330; *N*_*control*_ = 329). Four of these studies evaluated the impact of stem cell transplantation within 3 months of the incident stroke. The patients in these studies were more likely those with haemorrhagic stroke (*N*_experimental_ = 170; *N*_*control*_ = 136) than ischaemic stroke (*N*_experimental_ = 70; *N*_control_ = 70). On the other hand, seven studies that reported transplantation of stem cells more than 3 months post stroke had more patients with ischaemic stroke (*N*_experimental_ = 79; *N*_control_ = 116) than haemorrhagic stroke (*N*_experimental_ = 11; *N*_control_ = 7).

### Synthesis of results

#### Safety

Studies reported a varied period of safety follow-up to a maximum of 60 months following stem cell delivery. Safety events of particular interest are presented in Table 2. The most commonly reported adverse events included headache and fever, mostly self-limited and often related to the cell delivery procedures, particularly when administered via intracerebral/intrathecal routes.

**Table 2:** Early-phase stem cell studies in stroke: safety events

	Experimental (*n* = 461)	Control (*n* = 329)
Death	16	27
Tumours	5	0
Seizures	21	5
Recurrent stroke	8	1
Haematoma	5	0
Pain	3	0
Infections	11	9
Fever	19	1
Headache	14	0

Overall, 16 deaths were reported in participants receiving stem cell therapies. The cause of death was reported to be recurrent stroke (*n* = 3), infections (*n* = 3), cardiac causes (*n* = 8) and pulmonary embolism (*n* = 2). However, none of these events was ascertained as related to the therapy administered. The longest follow-up data published were from Lee et al. [19], who reported an adjusted hazard ratio (HR) of 0.344 (95% CI: 0.115–1.031, *p* = 0.057) for the mesenchymal stem cell group vs control for survival.

Twenty-one events of seizures were reported across 10 studies in patients receiving stem cells. The majority of these episodes were described as not related to the investigational therapy. These resolved with anti-epileptic treatment with no subsequent recurrence. Overall, five cases of tumours were reported (eccrine poroma (*n* = 1), lung cancer (*n* = 2), malignant melanoma (*n* = 2)). None of these were attributed to the stem cell therapy as patients had well-recognised risk factors for tumour (lung cancer; melanoma) in their past history prior to receiving stem cells.

### Effectiveness

#### Studies without a comparator arm

**Fig. 2:**
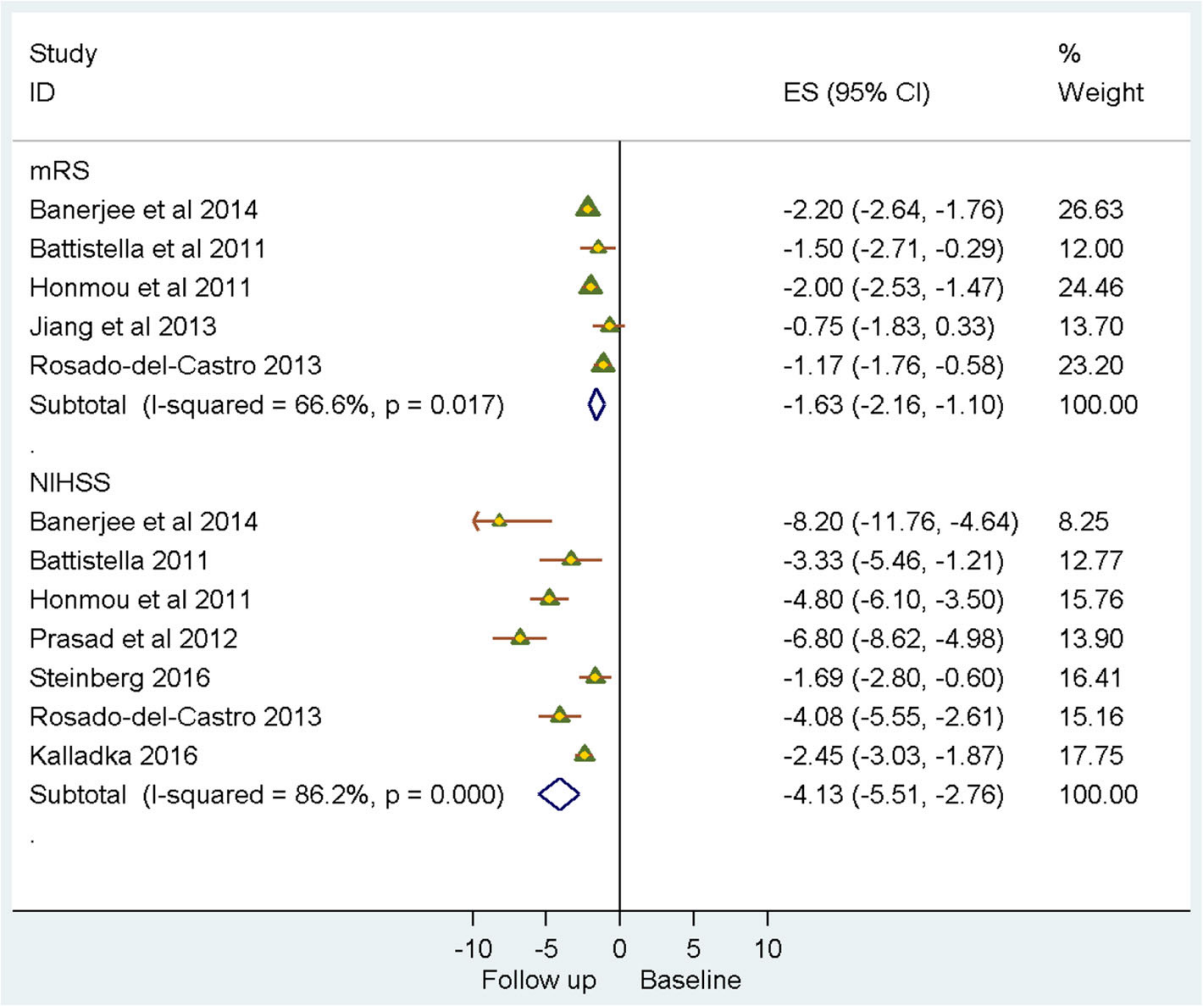
Single-arm studies: National Institutes of Health Stroke Scale (*NIHSS*)/modified Rankin score (*mRS*). *CI* confidence interval, *ES* effect size

**Fig. 3:**
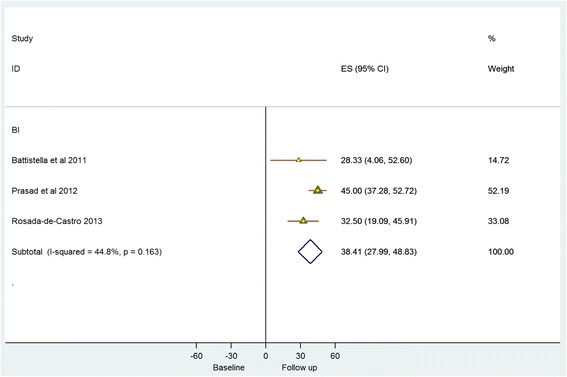
Single-arm studies: Barthel index (*BI*). *CI* confidence interval, *ES* effect size

Meta-analysis evaluated available data from eight single-arm studies that reported the impact of stem cell therapies at 6 months post treatment, on recognised validated body structure/impairment (NIHSS), activity (BI) and participation (mRS) measures (Figs. 2 and 3). NIHSS scores showed a modest decrease (SMD = – 4.13 (95% CI – 5.51 to – 2.76; *p* = 0.000)), although *I* ^2^ = 86.20% indicated significant heterogeneity across the studies. A similar, although numerically smaller, trend towards improvement was indicated by a decrease in mRS (SMD = – 1.63 (95% CI – 2.16 to – 1.10; *p* = 0.017); *I* ^2^ = 66.60%) and an increase in BI (SMD = 38.41 (95% CI 27.99–48.83; *p* = 0.163); *I* ^2^ = 44.80%)*.*

#### Studies with a comparator arm

**Fig. 4:**
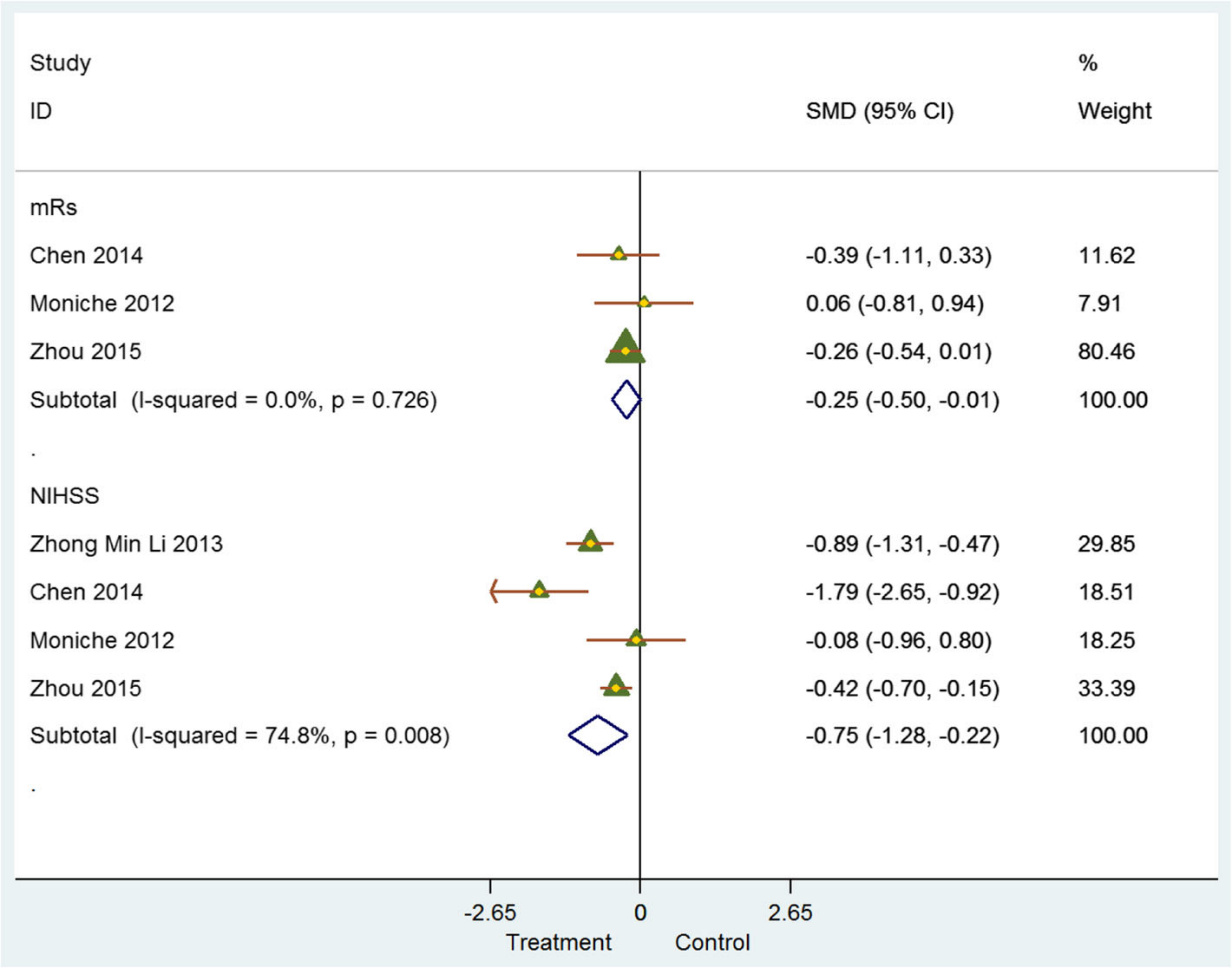
Controlled studies: National Institutes of Health Stroke Scale (*NIHSS*)/modified Rankin score (*mRS*). *CI* confidence interval, *SMD* standardised mean difference

**Fig. 5:**
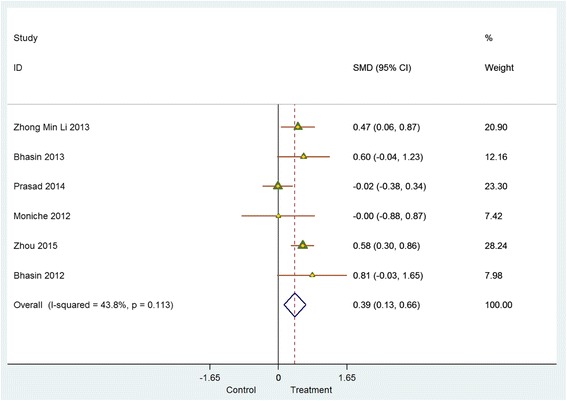
Controlled studies: Barthel index (*BI*). *CI* confidence interval, *SMD* standardised mean difference

Meta-analysis of data from six controlled studies that reported the impact of stem cell therapies on NIHSS, BI and mRS at 6 months post intervention revealed similar directional trends in the change of all three outcome parameters. However, the difference in effect size between experimental and control groups was very small (Figs. 4 and 5). NIHSS scores indicate a decrease (SMD = – 0.75 (95% CI – 1.29 to – 0.22; *p* = 0.008); *I* ^2^ = 74.8%). Similarly, mRS scores indicate a decline (SMD = – 0.25 (95% CI – 0.50 to – 0.01; *p* = 0.726)). BI scores demonstrated an improvement (SMD = 0.39 (95% CI 0.13–0.66; *p* = 0.113); *I* ^2^ = 43.80%).

### Assessment of risk of bias

**Fig. 6:**
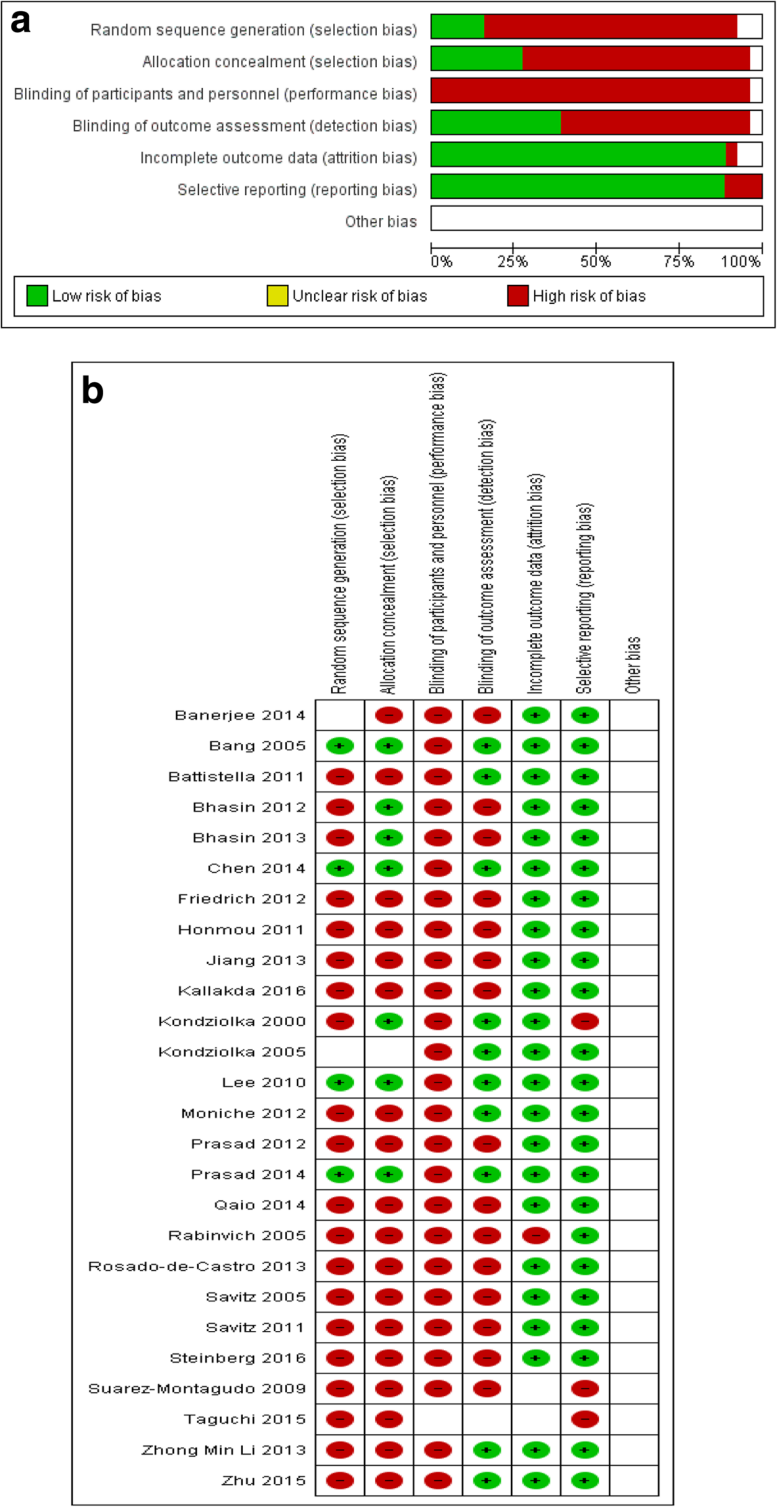
**a** Aggregate Risk of bias graph **b** Risk of bias summary

All studies had at least one or more source of bias (Fig. 6a, b).

#### Allocation

The sequence generation was adequate in 4/26 included studies. The method for sequence generation was not specified in three studies. The treatment allocation was concealed in only six out of 26 studies.

#### Blinding

None of the studies incorporated blinding of participants and study personnel. Ten out of 26 studies had blinding of outcome assessment.

#### Incomplete outcome data

Most studies reported having complete data for all included participants. In three studies, data on outcomes at 6 months were missing.

#### Selective reporting

All studies presented per-protocol data.

#### Other potential sources of bias

While selective publication of studies with significant results is regarded as a potential source of bias, there was no clear evidence of this in the current review. The impact of other sources of bias is equally hard to quantify.

### Additional analysis

Further subgroup or sensitivity analysis was not deemed feasible due to the small number of studies and limited data availability.

## Discussion

### Summary of evidence

The current review indicates a trend towards improvement across varied domains of functional impairment in patients with stroke given stem cell therapies. The quantum of improvement is small from studies that had a control comparator. A high level of heterogeneity was observed in both subgroups (with/without comparator). This may probably be due to the differences in cell types used and the route, dose and time of administration and other elements of the study design. An exploration of these factors as the source of the identified heterogeneity was not found to be feasible owing to the small number of patients involved. Therefore it is currently difficult to draw any meaningful conclusion about the most appropriate dosage and route of administration or the phase of stroke in which these therapies are likely to provide most meaningful benefit. Nonetheless, we believe that our study provides insights into the overall effect of stem cell therapy and provides a starting point for future research on this issue.

It is reassuring that the safety profile of these therapies has been reasonable, with no alarming signals to date, especially in relation to tumorogenicity. Most of the adverse events were self-limited and resolved spontaneously or with appropriate management. The events of most note were seizures, headache and events associated with procedures used for administering these therapies. In addition, all studies reported successful recruitment to target and successful administration of investigational therapy in study participants.

It is interesting to note that a greater proportion of more recent studies (since 2010) have investigated cells derived from an autologous cell source (15 out of 20 studies), whereas earlier studies had a similar number of studies with autologous (*n* = 4) and allogeneic (*n* = 5) sources of cells.

### Implications for clinical practice

There has been a steady increase in the number of studies published over the years (nine studies before 2010 and 20 studies published in the period 2010–2016). There is now an increasing body of evidence that administration of stem cell therapies in patients in different phases of stroke is feasible and encouragingly safe across different routes of administration. The key objective of early-phase clinical studies is to prove the concept and investigate preliminary safety of use in humans. To that end, our review and meta-analyses support the feasibility and safety of varied cell types delivered through different routes.

However, the strength of evidence to support effectiveness of these therapies is not robust. This is a challenge often seen in the early phase of development due to the small size of the studies typical at this stage. The direction of change indicates a potential benefit, which is consistent across both groups of studies (with or without a control comparison) and across outcome measures representing changes at the level of body function/impairment and those focused on daily activity and quality of participation in daily life. To gain stringent, clinically meaningful data as to potential benefit will require further research through well-designed phase 2/3 studies.

### Implications for research

With early-phase clinical studies investigating stem cell therapies reporting encouraging results, the field seems set to move into a phase where definitive effectiveness assessment becomes critical. The translational success with stem cell therapies in stroke, exciting as it may be, has posed questions that need addressing. The present review provides assurance for probable safety of cell therapies in patients with stroke and potential for further research. Our meta-analysis at this early phase of research is limited by significant heterogeneity in trial design and therapeutic strategies researched in these studies. However, the results of the analysis provide an early indication of potential benefit that should be explored through further research. This is necessary to avoid costly failures as in the past with neuroprotective interventions in stroke. Currently there is persistent ambiguity regarding clinical meaningfulness of interventions, despite increasing volumes of research data. Most importantly, the review reiterates the need to conduct adequately powered studies using well-characterised cell therapy products and investigating impact on standardised clinical recovery outcomes.

Stem Cell Therapies as an Emerging Paradigm in Ischemic Stroke (STEPS I/II/III) formulated recommendations on quality standards for pre-clinical and clinical research involving stem cell therapies [48, 49]. While these represent a much-needed framework to standardise regenerative research in stroke, most of the published studies had started prior to formulation of these guidelines. In fact, the challenges in design, feasibility and ethical aspects of these studies provided the impetus for the formulation of these recommendations to a significant extent. The ability to characterise the cells under investigation has been enhanced significantly with increased capabilities in immune phenotyping and molecular transcriptional profiling of investigational cell types. Recent studies have investigated more selective cell types as compared to earlier ones, which used naïve cells predominantly. These studies have referred to prior evidence of safety and impact on structural, functional and imaging parameters in rodent models in most instances. However, it is pertinent to note that the extrapolation of those findings may not always be straightforward. Numerous factors such as the differences in pathophysiological mechanisms of stroke between rodents and humans, the interplay of co-morbid conditions in humans and the current dearth of evidence for the impact of stem cells in animal models simulating such baseline characteristics need further investigation.

Two studies [18, 27] investigated cell disposition using cell labels (^99m^Tc and CD34-nano-iron complex) and reported variable homing and persistence of labelled cells in the brain. The study by da Fonseca et al. [18] also demonstrated distribution to other organs following IA administration. Numerous other studies have also reported extra-cerebral distribution of stem cells following IV/IA administration, which may have potential impact on eventual dosing and safety [18]. While these tracers might provide an indication for the initial distribution, they are limited in their ability to provide long-term information relevant to the lifetime of the implanted cells. Multimodal fate imaging using bioluminescence and fluorescence imaging with functional MRI has generated evidence for use for long-term viability and bio-distribution of stem cells [50]. This can potentially inform the period of safety follow-up considered adequate in early-phase research. For instance, a safety follow-up of 6 months is considered adequate for mesenchymal cell types, while a period of at least 1 year is recommended for most cell types [41, 49].

#### Study design—future considerations

STEPS III proposed that the inclusion criteria for phase 2/3 studies should be structured based on properties of the cell therapy under investigation, particularly if there are any safety signals detected in pre-clinical and phase 1 studies. While this is evidently sound science, it may be important to note here that the predominant proportion of phase 1 studies have failed to detect any obvious cell-dependent adverse events, specifically linked to a particular cell type. Exclusion of patients with significant co-morbidities might still therefore be required in the interest of safety, although this approach would limit extrapolation of eventual results to the general stroke population.

However, an issue of greater clinical relevance is the selection of trial endpoints in phase 2/3 studies. While recommendations from expert groups involved in stroke research have been highlighting the need to validate and adopt domain-specific endpoints, its true utility can only emerge if domain-specific endpoints are used to power these studies. There is increasing evidence validating the usefulness of domain-specific measures in quantifying and predicting potential trajectory of recovery [51]. However, for these to be more consistently utilised the following issues may need to be addressed.

The objective of phase 3 studies has traditionally been to prove effectiveness in as broad a proportion of the target population as is feasible considering evidence from pre-clinical and early-phase studies. Considering the heterogeneous nature of stroke, it may be more meaningful to investigate stem cells in specific areas of impairment caused by stroke. This may necessarily restrict patient inclusion to specific disability, but may provide more specific domain-centric outcome measures.

However, such study designs may face challenges from regulatory authorities who prefer studies to be powered to established global endpoints, discouraging the developers from choosing such endpoints. This is borne out by the present review, where most studies have reported temporal changes in NIHSS/mRS/BI. There is therefore an urgent need for researchers, clinicians and regulators to collaborate to review evidence on domain-centric outcome measures and provide guidance on how these could be incorporated in future trial design.

In addition, it is important to consider the unique pharmacodynamics of stem cells in the post-ischaemic microenvironment in the brain. The engrafted cells and consequent activation of paracrine pathways are potentially unique to the individual area and severity of ischaemic injury in a given individual. Even though the broad mechanistic direction of repair and plasticity may be similar across individual patients, the interactions between cells and target brain tissue are determined uniquely by an individual’s genotypic and phenotypic particulars. Thus it is reasonable to postulate that the individual’s natural course of recovery can impact the quantum of change seen in functional/structural outcomes [52]. Emerging data from the field of rehabilitation research have put forth an interesting concept of ‘the Maximum Proportional Recovery Rule’, which proposes that 70% of maximum possible change (i.e. spontaneous recovery) occurs in the first few months post stroke. Recent data support the applicability of this rule across different domain-specific impairments [53]. Potentially useful predictive algorithms that can plot a prognostic trajectory for this recovery by combining clinical, neurophysiological and neuroimaging data are being developed [54]. These algorithms, if validated across domain-specific populations, may provide a practical tool for stratifying patients into more homogeneous subgroups.

Because the effectiveness of rehabilitation and cell therapies may be driven by unique patient characteristics differentially, it may be pragmatic to consider delivery of stem cells accompanied by targeted rehabilitation as an ‘intervention package’ using a service delivery premise. The measures of effectiveness with such restorative interventions are often continuous variables that require definition of the minimal clinically important difference (MCID) that is acceptable to prove benefit. The necessary next question is whether the conventional randomised controlled design is the ‘best fit’ for generating data to inform clinical practice in this fast evolving field.

Cluster randomisation with factorial design to incorporate multiple interventions (i.e. stem cell transplantation and rehabilitation) may be a pragmatic design to consider [55]. Study design can incorporate clusters of patients defined by domain-specific impairment receiving targeted, standardised rehabilitation in addition to stem cells. The effectiveness can then be assessed in terms of quantum of change on domain-specific endpoints.

An equally important area of research is defining the time points in stroke evolution more consistently in line with emerging tissue and imaging evidence. The chronic phase of stroke represents the area of greatest unmet medical need. However, it is interesting to note that while there are increasing data from rehabilitation and stem cell research in chronic stroke, the clinical determination per se of stroke as ‘chronic’ is heterogeneous, making any comparison/pooling of data difficult.

### Limitations

The findings of the present review and meta-analysis should be examined in the light of a number of study limitations. First, high levels of heterogeneity were observed across studies, which differed in terms of therapeutic characteristics such as route of administration, timing after stroke and dose. We acknowledge this limitation and therefore have been conservative in our pooling and in our analysis techniques. Unfortunately, there were too few studies to explore these factors as potential sources of heterogeneity either through subgroup analysis or meta-regression. As a result, we are unable to draw any inferences about the optimum dose or route of administration. Such investigations may be more feasible as further studies appear in the literature.

Second, most of the studies included had small numbers of patients which may have resulted in small study effects, particularly in single-arm studies where the samples rarely reached double figures [56]. Small sample size is expected in early-phase research but this made any additional subgroup analysis unfeasible. Third, potentially relevant studies had to be excluded because of the lack of published information and non-availability of the additional information on request. Lastly, language bias remains an issue as we searched only English-language databases and journals.

## Conclusions

This review and meta-analysis provides further evidence for the safety and feasibility of cell therapies for stroke. There is reasonable evidence to suggest feasibility, safety and potential effectiveness of these therapies. In view of the heterogeneity of disease per se and the nascent characterisation of therapies, the review poses important questions that are critical to translational success. Further progress in this field will require execution of phase 2/3 clinical trials with study designs that ensure homogeneity of stroke characteristics, potentially with domain-specific characterisation of disabilities and targeted provision of rehabilitation and with appropriate robust control to answer the fundamental question of effectiveness.

## Additional file

Additional file 1: Search Strategy. (DOCX 20 kb)
