# Supplementary material for: Safety and effectiveness of stem cell therapies in early-phase clinical trials in stroke: a systematic review and meta-analysis
Source: Stem Cell Res Ther. 2017 Aug 30;8:191. doi: 10.1186/s13287-017-0643-x (PMC5577822; doi:10.1186/s13287-017-0643-x)
Supplement: Additional file 1: — Search Strategy. (DOCX 20 kb) [file 13287_2017_643_MOESM1_ESM.docx]

**Additional file 1: Search Strategy**

*Logic Grid*

| AND AND AND AND | | | |
| --- | --- | --- | --- |
|  | Cell therapy | Delivery method | Cell source |
| Stroke* OR  CEREBROVASCULAR ACCIDENT*[tw] OR  CEREBROVASCULAR INSULT*[tw] OR CEREBRAL INFARCT*[tw] OR cerebral ischem* [tw] OR CEREBRAL HEMORRHAGE*[tw] OR INTRACRANIAL HEMORRHAGE* [tw] OR Intracranial embolic*[tw] | stem cell*[tw] OR Mesenchymal stem cell*[tw] OR bone marrow stem cell* [tw] OR peripheral blood stem cell*[tw] OR haematopoietic stem cell*[tw] | transplant* OR implant* OR infusion* OR neurosurg* | autologous* OR allogeneic* |

*PubMed*

(CEREBROVASCULAR TRAUMA*[TW] OR Stroke* OR Stroke[MH] OR CEREBROVASCULAR ACCIDENT*[tw] OR CEREBROVASCULAR INSULT*[tw] OR CEREBRAL INFARCT*[tw] OR cerebral ischem* [tw] OR CEREBRAL HEMORRHAGE*[tw] OR INTRACRANIAL HEMORRHAGE* [tw] OR INTRACRANIAL HEMORRHAGE[MH] OR Intracranial embolic*[tw]) AND (stem cell*[tw] OR STEM CELLS[MH] OR IPS Cell*[TW] OR PROGENITOR CELL*[TW]) NOT ((ANIMALS[MH] OR ANIMAL*[ALL] OR RAT[ALL] OR RATS[ALL] OR MICE[ALL] OR MOUSE[ALL] OR SHEEP[ALL]) NOT (HUMAN[ALL] OR HUMANS[ALL])) NOT REVIEW[PT]

*EMBASE*

stroke* OR cerebr* NEXT/2 (insult* OR injur* OR insufficienc* OR trauma* OR infarct* OR hemorhage* OR haemorrhage* OR isch* OR accident* OR lesion* OR embolic*) OR brain NEXT/1 (hemorhhage* OR haemorrhage* OR embolic*) AND ([cochrane review]/lim OR [systematic review]/lim OR [controlled clinical trial]/lim OR [randomized controlled trial]/lim OR [meta analysis]/lim) AND [english]/lim AND ([young adult]/lim OR [adult]/lim OR [middle aged]/lim OR [aged]/lim OR [very elderly]/lim) AND [humans]/lim AND ([embase]/lim OR [medline]/lim) AND [2005-2016]/py AND (stem OR progenitor OR ips) NEXT/1 cell* AND [english]/lim AND ([young adult]/lim OR [adult]/lim OR [middle aged]/lim OR [aged]/lim OR [very elderly]/lim) AND [humans]/lim AND ([embase]/lim OR [medline]/lim) AND [2005-2016]/py

*Web of Science*

(STROKE*) OR(BRAIN NEAR/1 (HEMORHHAGE* OR HAEMORRHAGE* OR EMBOLIC*)) OR (CEREBR* NEAR/2 (INSULT* OR INJUR* OR INSUFFICIENC* OR TRAUMA* OR INFARCT* OR HEMORHAGE* OR HAEMORRHAGE* OR ISCH* OR ACCIDENT* OR LESION* OR EMBOLIC*)) AND ((STEM OR PROGENITOR OR IPS) NEAR/1 CELL*) AND (HUMAN*) NOT (MICE OR RAT OR RATS)

*Cochrane Central Register of Controlled Trials*

STROKE* or CEREBR* next (INSULT* or INJUR* or INSUFFICIENC* or TRAUMA* or INFARCT* or HEMORHAGE* or HAEMORRHAGE* or ISCH* or ACCIDENT* or LESION* or EMBOLIC*) or BRAIN next (HEMORHHAGE* or HAEMORRHAGE* or EMBOLIC*):ti,ab,kw

AND (STEM or PROGENITOR or IPS) next CELL*

*SCOPUS*

( ( TITLE-ABS-KEY ( cerebr* PRE/2 ( insult* OR injur* OR insufficienc* OR trauma* OR infarct* OR hemorhage* OR haemorrhage* OR isch* OR accident* OR lesion* OR embolic* ) ) OR TITLE-ABS-KEY ( brain PRE/1 ( hemorhhage* OR haemorrhage* OR embolic* ) ) OR TITLE-ABS-KEY ( stroke* ) ) AND DOCTYPE ( ar ) AND PUBYEAR > 2004 ) AND ( TITLE-ABS-KEY ( ( stem OR progenitor OR ips ) PRE/1 cell* ) AND DOCTYPE ( ar ) AND PUBYEAR > 2004 ) AND ( ( TITLE-ABS-KEY ( human* ) AND NOT TITLE-ABS-KEY ( mice OR rat OR rats ) ) AND DOCTYPE ( ar ) AND PUBYEAR > 2004 ) AND ORIG-LOAD-DATE AFT 20160605 AND ( LIMIT-TO ( LANGUAGE , "English" ) )
